# National pharmacy associations in the commonwealth: current scenario and future needs to maximise effective contributions of the pharmacy profession towards universal health coverage

**DOI:** 10.1186/s40545-021-00396-7

**Published:** 2021-12-27

**Authors:** Ayesha Iqbal, Victoria Rutter, Gizem Gülpınar, Manjula Halai, Briella Awele, Rasha Abdelsalam Elshenawy, Claire Anderson, Rabia Hussain, Amy Hai Yan Chan

**Affiliations:** 1 Commonwealth Pharmacist Association, London, UK; 2 Division of Pharmacy Practice and Policy, School of Pharmacy, University of Nottingham, Nottingham, NG7 2RD UK; 3 Department of Pharmacy Management, Faculty of Pharmacy, Ankara University, 06560 Ankara, Turkey; 4 Department of Clinical and Pharmaceutical Sciences, FADIC School of Antimicrobial Stewardship, University of Hertfordshire, Hatfield, WD19 6LN UK; 5 Department of Social & Administrative Pharmacy, School of Pharmaceutical Sciences, Universiti Sains Malaysia, 11800 Penang, Malaysia; 6 School of Pharmacy, Faculty of Medical and Health Sciences, University of Auckland, Grafton, 1023 New Zealand

**Keywords:** Commonwealth Pharmacy Association, Pharmacy workforce, Pharmacy practice, Universal Health Coverage, Sustainable Development Goals

## Abstract

**Background:**

The Commonwealth Pharmacists Association (CPA) is a charity representing pharmacists across the Commonwealth, with the vision of empowering and collaboratively develop the profession and fully utilise the potential of pharmacists to strengthen health systems through supporting better access to, quality and use of medicines and related services. Commonwealth comprises predominantly of low- and middle-income countries, where limited data often exists in pharmacy practice. There is a recognised need across the Commonwealth to focus on developing, implementing and fully utilising pharmacy professional services to progress universal health coverage and achieve the sustainable development goals, particularly in low and middle-income countries; however, currently a knowledge gap exists in understanding the national priorities in Commonwealth nations. CPA is ideally positioned to access to these nations. The aim of this study was thus to explore the priorities and focus areas of NPAs across the Commonwealth and create evidence for a needs-based approach to inform the support that the Commonwealth pharmacists association can collaboratively and strategically provide to its members to progress towards shared goals.

**Methods:**

Data were collected virtually on Zoom by conducting interviews using a semi-structured interview guide developed for this study with CPA councillors representing NPAs or their equivalents if no official body existed. An inductive, reflexive, thematic analysis was performed for data analysis.

**Results:**

In total, 30 councillors were interviewed from 30 low- and medium-income countries. The three main overarching priority areas identified across respective Commonwealth nations developing extended pharmacy services, improving pharmacy education, and developing and redefining the role of NPAs.

**Conclusions:**

This novel study highlights the collective priorities for the pharmacy profession across the low and middle-income countries of the Commonwealth and the urgent need for supporting NPAs around the three identified overarching priority areas. The mapped-out priorities will inform an evidence-based approach for the CPA to better support NPAs in their mission through advocacy and practitioner development, to fully harness pharmacists’ unique skill set and maximise their contribution to progressing universal health coverage.

## Background

Universal health coverage (UHC) means that all people and communities across the world can have access to quality medicines and medicine services without exposing the end user to financial hardship [1]. Following UHC aims, many nations across the world have incorporated the philosophy of UHC in their national health policies and translated it into achievable targets in terms of increasing public access to equal, accessible, affordable and sustainable medicines and medicine services [2–4]. The United Nations sustainable development goals (SDGs) support the overall goals of UHC and aim to obtain “access to safe, effective, quality, and affordable essential medicines and vaccines for all” [5]. Access to affordable, quality-assured essential medicines is crucial to reducing the financial burden of care and improving population health worldwide. An ideal health care system adapted to achieving UHC goals to increase public access to medicines would need to consider developing, collaborating and improving health systems through the development of quality health services and workforce as well as expanding coverage of affordable medicine and overall health care [5]. Currently, policy development inspired by UHC agendas is core focus areas of international and national; governments and health organisations [6, 7].

An integral component of UHC goals ensures that every person has the right to quality affordable services and quality essential medicines without incurring additional costs to people and health care systems [8, 9]. Chronic diseases and their management strains health systems and individuals across the world because of limited health workforce [10]. A third of the world’s population lacks access to essential quality medicines and services [11]. In many low and medium-income countries (LMICs), medicines are unaffordable for people who need them. There is a need to increase access to primary care services, access to affordable quality medicines and improve patient outcomes especially in the management of chronic conditions. Majority commonwealth nations are LMICs and there is unavailability of access of quality medicines and medicine services which becomes of eminent concern for the management of chronic disease and their rational management [8, 9, 12].

Pharmacists are one of the most easily accessible health care providers and are often the first health professionals that the public refers to for advice and care in health care needs. The concept of pharmaceutical care was introduced in 1990 and since then have been continuously developing and evolving to shift from product-centred to patient-centred services and provide an array of numerous services and opportunities, which can have an enormous impact on public health [9]. As the global agenda shifts towards addressing the SDGs, pharmacists are ideally placed to collaborate with other health care members as acknowledged medicine experts to help strengthen health care systems to achieve the third SDG of “good health and well-being for all” [13, 14].

The International Pharmaceutical Federation (FIP) and United Nations recognises the role of pharmacists in achieving SDG and UHC goals [15]. Pharmacists' involvement in ensuring quality medicines and medicine services to achieve optimal patient safety has been crucial in high income countries, [16, 17]. In high income countries, pharmacists are increasingly recognised and engaged in public health activities such as disease management and prevention, medicine optimisation and reviews, extended clinical and social services, immunisation and policy development. Studies have shown that pharmacists with more comprehensive responsibilities have lowered total costs and achieved improved quality of care outcomes by health care systems, particularly related to chronic conditions [18]. However, there is a lack of utilisation of pharmacy services in many LMICs across the Commonwealth (and beyond) as compared to high income countries. Increased health care services utilisation will not result in better outcomes if pharmacy services are not adequately developed to support health care systems, especially if the quality of medicines and pharmaceutical services is low [17]. FIP recognises the critical role the pharmacy profession and identifies a particular need and opportunity for pharmacists to be effectively utilised to increase access to safe, quality medications that can save lives. From medication safety and the responsible use of medication to supply chain management and immunisations, pharmacists can utilise their medication expertise not only for addressing local gaps in care, but also for broader global health efforts [14].

The Commonwealth Pharmacists Association (CPA) is a registered charity that is uniquely positioned to advance health, promote wellbeing and improve medicines-related education for the benefit of the people of the Commonwealth. This voluntary network of member states encompasses 1/3 of the global population including many LMICs. CPA is an accredited organisation of the commonwealth and an active member of the Commonwealth Health Profession’s Alliance (CHPA), which advocates for all aspects of health to national policymakers and Commonwealth governments. Regarding achieving the SDGs and UHC goals, the CPA’s mission is to promote and ensure safe and effective medicines use to improve health and well-being throughout the Commonwealth nations. CPA’s unique position as a civil society organization provides a network and centralised platform that could potentially support all Commonwealth nations to achieve UHC goals through collaborative working, advocacy and support [19]. As an accredited organisation of the Commonwealth, the CPA supports advancement of Sustainable Development Goal (SDG3) and progress towards UHC through the development of safe and effective systems of medicines management, healthier lifestyles, and the reduction of health inequalities. The CPA achieves this through building strong collaborative networks, partnering with member organizations such as National pharmacy associations (NPAs) to improve the quality of pharmacy practice and creating platforms for the dissemination of knowledge about pharmaceutical sciences and professional practice. The CPA in line with SDG 17 (partnership) has been working to support NPAs throughout the Commonwealth towards national-level development and implementation of these goals. The CPA is in official relations with WHO and has an approved collaboration plan.

Utilising the pharmacy workforce to cater to health care needs becomes even more meaningful when considering the shortfall of 18 million health workers by 2030 that is projected by WHO and the amplification of this problem in LMICs [20]. CPA has currently 54 registered members nations [21] and aims to support member nations through their national pharmacy bodies to achieve the respective national and international UHC goals and to provide equal and effective services for the health care needs of their respective populations.

Due to the diverse nature of pharmacist representative bodies used across Commonwealth nations, in this study, we have used the term “NPAs” to represent pharmacy professional bodies. This encompasses pharmacy organisations, pharmaceutical societies, and pharmacy associations in Commonwealth nations, respectively. This, thus refers to any official pharmacy representative body in any Commonwealth nation, which is associated with the development and improvement of pharmacists and the pharmacy profession. We also use the term ‘independent pharmacy regulator’ for pharmacy councils which regulate the pharmacy profession and is a separate entity to a medicine regulator, which in a broad context regulates the quality, access and pricing of medicines.

Recognising the need and developing appropriate policies and services to account for local public health requirements as well as fulfilling international global health agenda, are essential functions of NPAs to enable operationalisation of policies and services in the unique health systems, respectively [22–24]. NPAs planning and development are critical if we are to achieve UHC. However, the plans of NPAs for the development of the pharmacy profession are not always aligned according to local needs. Taking this into account may allow CPA to identify and support member organisations to in their planning around achieving UHC goals.

Thus, the aim of this study was to explore the priorities and focus areas of NPAs across the Commonwealth for developing the pharmacy profession to inform how the support that needs to be provided to strengthen their unique health care systems to achieve UHC and SDGs goals.

## Method

### Study design

Due to the nature of the research aims, and lack of existing research on this topic, we chose a qualitative, exploratory study design to address the question under study. The qualitative design adopted for this study enabled flexibility and an in-depth exploration of representative participants’ perspectives and intentions, which survey-based research could not achieve [25]. Data was collected virtually by conducting interviews using a semi-structured interview guide with councillors and representatives of NPAs Commonwealth nations to facilitate a detailed exploration of their perspective and priorities.

### Participants and settings

We used a purposive sampling strategy to recruit CPA councillors representing NPAs or their equivalents if no official body existed who were actively engaged in the development and revision of new pharmacy services in their respective nations. Study participants were invited to take part in this study by contacting them via their e-mail addresses, already present in CPA database at the time of membership. All CPA member organisations have previously given consent to be contacted by the CPA in accordance with general data protection data regulation (GDPR) for improvement, evaluation and development purposes as part of the membership agreement.

The membership and communication officers at CPA (AI, TH) contacted every nations’ councillor or representative, three times via email in case of no response to initial email(s). Interviews were conducted at a mutually convenient time using Zoom®. These interviews were an essential part of the CPA’s quality service improvement and engagement plan at CPA's, 50th birthday and involved a virtual tour and to scope the NPAs for recent achievements and priorities for the pharmacy profession in their country, the former of which is beyond the scope of this article.

No incentives were provided for participation in the study. The interviews were audio recorded and later transcribed verbatim after obtaining participant’s consent before the start of the interview.

### Data collection

All councillors from LMIC members (*n* = 44) were invited to participate in this virtual tour. Those nations, which did not respond to the initial study invitation, were contacted again after 2 and 4 weeks, respectively. High income Commonwealth countries were also contacted, and their responses were recorded, but were excluded from data analysis for this study. All consenting councillors were interviewed by the CPA team between January 2020 and December 2020. A team of two interviewers; VR and MH conducted all interviews; and were joined by the author AI during the first eight interviews to review and revise the interview guide if needed and to familiarize AI with the nature of data being generated.

The interviews were conducted in English. Data was collected using a semi-structured interview guide designed in line with the aims of this study, as shown in Table 1. The interview guide was pilot tested for face validity by 2 CPA internal members. The interviews by the CPA research team were exploratory to focus on understanding the NPAs priorities. The interview guide was used as an exploratory guide and mainly focused on exploring; current achievements of the NPAs or professional bodies and future priority areas regarding NPAs vision and policies to develop and/or upgrade pharmacy profession and practices to achieve UHC and SGD goals, as well as exploring facilitators and barriers in participants’ respective nations. The interview guide was reviewed after the first three interviews, but no changes were made to it. The interviews were conducted, concurrently with the analysis and the thematic saturation was achieved after 27th interview. However, the interviews continued until all consenting councillors from LMICs of Commonwealth nations were interviewed.

**Table 1:** Semi-structured interview guide

Sr. No.	Questions
1	What are your three main priorities/goals to progress pharmacy profession in the country as a NPA to achieve UHC goals in the next 5 years?
2	What are the barriers/deficiencies in achieving these specific goals?
3	What steps have been taken internally to overcome these barriers?
4	Any barriers or initiatives taken so far?
5	What are your expectations from CPA in helping or assisting you overcome these barriers?
6	Anything you would like to share that seems important as a NPA priority

### Data analysis

**Fig. 1:**
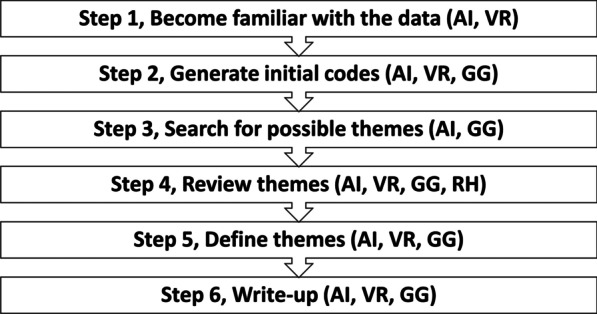
Steps conducted during an inductive reflexive, thematic analysis

An inductive, reflexive, thematic analysis [26, 27] was performed for data analysis. The six-step analysis framework by Braun & Clarke was used as a guiding methodology for performing a reflexive, thematic analysis, as shown in Fig. 1. Our analysis method was also influenced by the constant comparison method of grounded theory and was followed in the later stages of the analysis to compare independent themes amongst the three researchers (AI, GG, VR) and a field expert (RH) in carrying out the reflexive thematic analysis [17].

All the interview recordings were de-identified by assigning a protocol number as follows: “C” (for councillors) and the sequence number of the interviewee and their geographical location (e.g., C1-Africa) and were transcribed verbatim. We used NVivo qualitative analysis software v12 for coding (QSR International Pty Ltd. Version 12, 2019). The verbatim translations in the transcript were later edited intelligently [28]. All the analysis of the transcribed data followed a semantic approach, as shown in Fig. 1 [26]. This provided step 1 “familiarity” in thematic analysis with the data. In step two, the data was then coded “line by line “, interpreted and discussed by all three authors. In step 3, the similar codes of data were grouped together into look alike sub-themes and discussed. In Step 4, the themes were cross validated to avoid duplication of subthemes into individual themes. The data was independently coded by three authors AI, GG, VR to reduce individual bias and the research team met every 2 weeks to discuss the potential reflection of ideas and themes during the analysis. A field expert from a LMIC, RH was also approached and asked to review the codes and themes to introduce rigor and reduce the researcher team’s own influence and bias. In step five, three major themes were reviewed and seemed to fit the overall criteria of this study after the consensus of all authors. The entire research team reviewed and commented on the final set of themes. In the sixth final step, a detailed report was drafted by AI, VR and GG to give an overview of the exploratory findings across Commonwealth nations. At all stages, the authors (AI, GG and VR) stayed in constant communication and overall, the data analysis followed the reflexive, thematic analysis framework and grounded theory constant comparison method. The research was intended to be descriptive and exploratory rather than concerned with developing a theoretical framework due to the nature of the research aims of this study.

## Results

A total of 30 councillors were interviewed from 30 LMIC member nations (out of a total of *n* = 44 members contacted), giving a response rate of 68.1 percent. The geographical distribution of participants is represented in Table 2.

**Table 2:** Geographical location of participating low- and middle-income countries [29]

Continent		Low-income countries	Middle-income countries
Africa	Central	2	1
	East-Central	3	–
	Eastern	4	2
	Western	1	1
	Southern	–	2
Asia	South	–	2
	South–East	–	3
America	Northern	–	4
	Central	–	3
Pacific	South–West	–	2

**Fig. 2:**
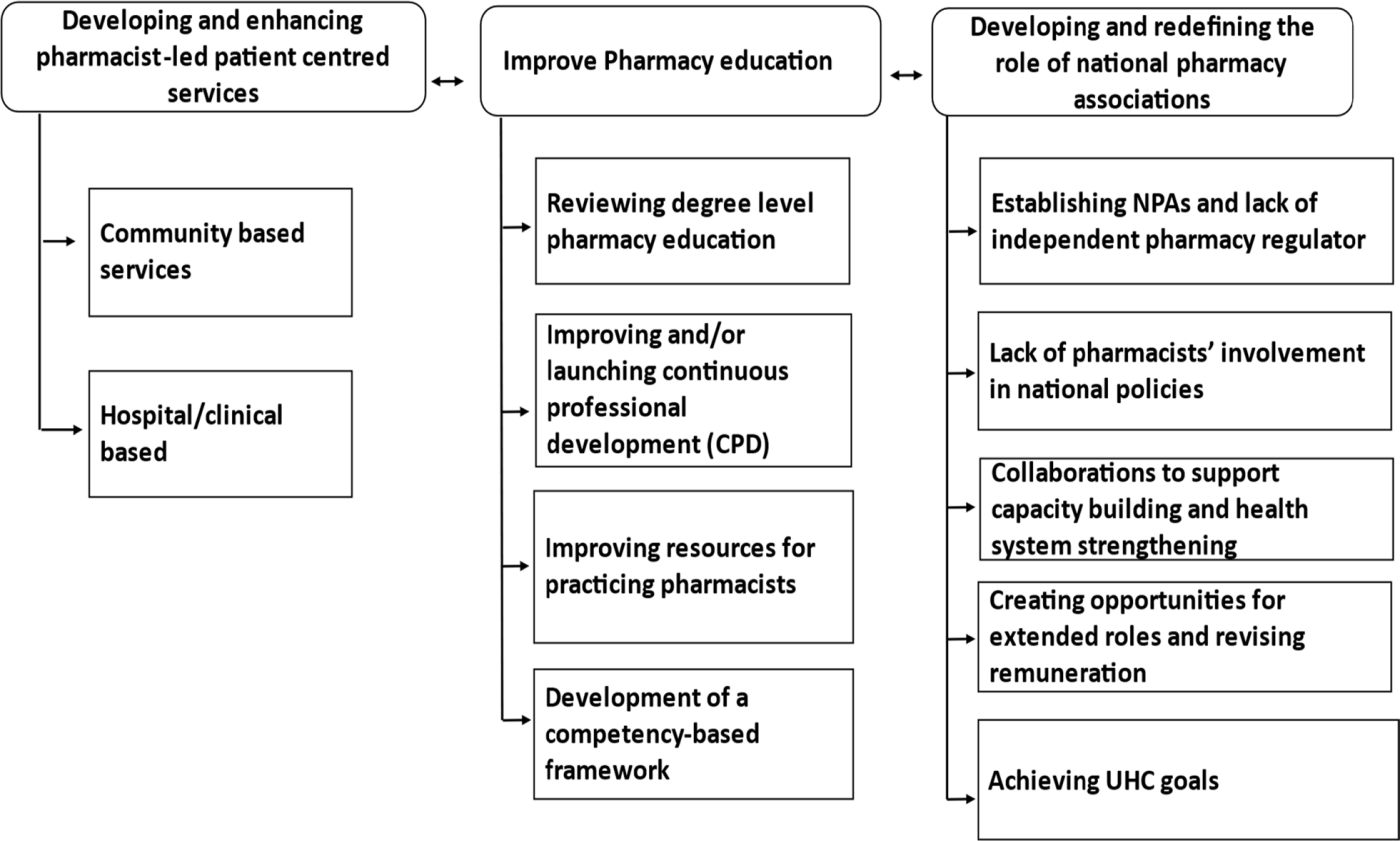
Themes and subthemes of priorities for commonwealth nations

Interviews lasted from 60 to 120 min on average. During the reflexive, thematic analysis three major overarching themes were identified which were: (i) developing and enhancing pharmacist-led patient centred services; (ii) improving pharmacy education; and (iii) developing and redefining the role of NPAs. Figure 2 shows an overarching diagram representing the themes and their underlying sub themes.

### (i) Developing and enhancing pharmacist-led patient centred services

Recent evidence from high income countries has supported the integration of pharmacists in the health care systems. However, councillors shared that pharmacists in LMICs from the Commonwealth are still being utilised for conventional dispensing roles and the health systems currently lack extended pharmacist services to support UHC and SDG goals. Councillors also indicated NPAs in LMICs across the Commonwealth are currently focussed on developing, launching and improving pharmaceutical care services to help achieve UHC goals of increased assess to medicine services in two interconnected sub-themes: (a) community sector and (b) hospital sector.

#### (a) Community-based pharmacy services

Councillors felt that many LMICs community pharmacy sectors still lack patient-based services. The councillors identified a critical need to develop community pharmacy-based service enhancements and NPAs are prioritising developing this sector to help train pharmacists and utilise them provide clinical services in the community pharmacy sector.

*“Community pharmacists in the private sector … It is quite lagging with how pharmacists respond to patients. We're fairly poor at training (pharmacists) around minor ailments, and practical stuff around communications, we need to focus on developing the role of community pharmacists”* South Asia—C15

NPAs are also focusing on developing specialised community pharmacy-based services in antimicrobial resistance (AMR), non-communicable disease management (NCDs), countering medicine misuse and delivering vaccinations, which councillors suggested could help achieve better and optimised patient outcomes.

*“The main hurdle is to combat the NCDs (non-communicable diseases): diabetes, lipid problems, and cardiac disease. Extremely ongoing and rising year to year, we need pharmacists (community) to help with it”* Eastern Africa—C11

*“It might be interesting to see how we can get community pharmacists. involved in Anti-microbial stewardship... we are working on it”* Western Africa—C1

#### (b) Hospital-based pharmacy services

The concept of pharmaceutical care has been adapted by advanced nations. However, the councillors shared that in resource limited nations pharmacists are still mainly working in conventional dispensing and procurement roles. The councillors felt that NPAs should focus on adapting the global strategy of clinical pharmacy and building standard pharmacy practices around the core concept of pharmaceutical care. In addition, councillors mentioned that the NPAs should be updating and redefining their roles to better support the development of new clinical roles for pharmacists.

*“Getting pharmacists more involved in hospital practices is something that will be extremely exciting for us, the advanced countries do it, why not us”* Western Africa—C1

*“In terms of clinical pharmacy, the majority of hospitals still do not utilise the skills of clinical pharmacists, they (pharmacists) are not facing patients”* Eastern Africa—C2

Councillors felt that due to lack of clinical pharmacist involvement, the burden of health care falls on other healthcare members such as nurses. This is also affecting the quality of overall health services.

*“The areas clinical pharmacy would be useful for is like oncology! But they are not yet found in these places, so, it’s female nurses who have to do the mixing and the dispensing instead.”* Eastern Africa—C11

*“It’s been an aspiration to have a fully functional clinical pharmacy program. We have not been able to make it real, we have had obstacles with staff (pharmacists), but it is something that has importance to us. We’ve always been interested in clinical pharmacy.”* The Americas—C8

### (ii) To improve pharmacy education

The overarching theme of “improving pharmacy education” includes three sub-themes according to the councillors’ expressions reflecting a need to (a) reviewing degree level pharmacy undergraduate education (b) improving and/or launching continuous professional development (CPD) (c) improving educational and practice resources for pharmacists and (d) development of competency-based frameworks.

#### (a) Reviewing degree level pharmacy education

To help improve pharmacy education, some councillors stated a priority of upgrading skills and educating academics so that teaching and curricula is up to date and reflects current clinical practice needs and competencies.

“The academia should prepare pharmacists to practice effectively; the teachers need to be exposed more to the clinical practice and contact with more patient cases in order to be able to provide an applied academia” South East Asia—C9

#### (b) Improving and/or launching continuous professional development (CPD)

Most of the councillors perceived that there is a need for improving and/or launching continuous professional development (CPD) across Commonwealth nations to increase the involvement in clinical settings and to progress the pharmacy profession in terms of need based practices, specially to prepare them for new practice-based roles.

*“The CPD is a priority for us, especially for postgraduate involvement in clinical services. Supporting us in reference materials and helping us to really strengthen our society and association is very important”* Western Africa—C17

*“The non-communicable diseases in respective local geographical locations such as diabetes and hypertension need more improvement, technical tools and materials to improve the (services by) pharmacists.”* The Americas—C8

CPD should include a range of topics, spanning clinical elements but also soft skills, including leadership training to facilitate pharmacists leading on service development.

*“Social, administrative and leadership pharmacist who received their fellowship qualification in the college(s) of pharmacy should have a leadership training skill and how to do a business plan, additionally, it is important to improve their areas of pharmacy practice and should be able to put a plan for what they want to achieve in the future as a leader”* Western Africa—C9

#### (c) Improving resources for practicing pharmacists

Councillors shared that they would like to continue benefitting from CPA to receive practical materials for education, such as books; however, expanding the CPA support to access electronic resources would be ideal for training of pharmacists in a wide geographical location to improve pharmacists’ skills in pharmacy practice services.

*“The need for the membership renewal in PharmAid, because the registration has finished four years ago and pharmacists need more technical and electronic resources to be updated in the pharmacy practice, additionally, we need access for the library of the most important e-resources.”* The Americas—C8

“We have to look at better ways on how to conduct CPD online. How do we tap into using CPD to have different various sessions, or to catch up on sessions for pharmacists who are unable to attend the first time? We need to be progressive in thinking of how to make CPD more interactive with the whole community” East-Central Africa—C6

Lack of electronic resources and systems have also been a barrier following the impact of COVID-19 pandemic and the adjustment of health care systems to minimise the impact to patients. The NPAs focused on moving some pharmacist-based services online, which proved to be a difficult task to achieve remotely due to lack of electronic systems.

#### (d) Development of a competency-based framework

Developing competency frameworks according to local needs in Commonwealth nations will help pharmacists achieve the educational skills and professional standards according to individual nation context, needs and developing a career pathway.

*“Only started registering pharmacists 6 years ago, not reached the level of the GPhC registration, and still low level of qualifications, but need to develop the resource for the pharmacy competency”* The Americas—C12

*“We do have access to websites but how do we make sure that the pharmacists are learning the correct things or if they are learning at all? so we need a competency framework for re-registration every year” The* Americas—C12

*“We need to focus on our own competency framework for pharmacy practice to guide pharmacy training and practice”* East Africa—C2

### (iii) Developing and redefining the role of NPAs

The need to refocus and redesign the scope, actions and responsibilities of NPAs is a priority of many of the Commonwealth nations to facilitate the development of the pharmacy profession as well as to provide support to pharmacists via flaws and legislation that support professional development. The councillors also felt that there should be separate a pharmacy (profession) independent regulator as well as a medicines regulator to develop and achieve professional standards as well as to maintain and increase access to affordable and quality medicines to aid in achieving UHC goals. Five overarching sub-themes for the development of NPAs roles were identified and are presented below.

#### (a) Establishing NPAs and lack of independent pharmacy regulator

Commonwealth nations, which do not yet have a registered NPA or an independent pharmacy regulator or a pharmacy council, felt that a lack of professional body might be a barrier for advocating for pharmacists’ rights as well as regulating the profession. Councillors expressed that they would like to develop these NPAs to help support the pharmacists, pharmacy education and secure benefits for pharmacists as well as develop independent pharmacy regulators to help regulate the profession.

*“We don't have a pharmacy council, yet we are trying to form the council, otherwise we don’t have any standards”* West Africa—C1

In this study, Commonwealth councillors, especially from the African nations, mentioned that they perceive their NPAs are solely concerned with medicine sale, ensuring quality of medicines, accessibility, distribution, and pricing regulation and are essentially lacking in support for capacity building of pharmacy workforce and developing or revising professional services. Councillors also strongly perceived that they were currently lacking in voice and representation on the national levels due to the absence of NPAs. Councillors also felt a strong need to have a distinctive medicine regulatory body to regulate the sale of medicine and product related issues, along with a separate body as an independent pharmacy regulator that can widely focus on pharmacy profession standards and regulation.

*“We feel that we need to have a separate pharmacy council and medicines and health product regulators. Both internal and external advocacy is needed in respective domains.”* East Africa—C2

#### (b) Lack of pharmacists’ involvement in national policies

Following from the lack of NPAs one of the common barriers that NPA councillors perceived in context of advocating for pharmacists and pharmacy rights on national levels, was having physicians representing pharmacists at national policy levels instead of pharmacists.

*“The main issue is that there's no pharmacy bill. We don't have these legislations, so it needs to be approved however there is a barrier due to doctors being in control of parliament and hijacking the pharmacy bill.”* South East Asia—C3

*“It is almost becoming policy and written that every health care institution must be headed by a medical doctor. We're thinking this should not be so, management should go to the right person whether he's a doctor or pharmacist.”* Western Africa—C4

Councillors across Commonwealth nations indicated that pharmacists should be included in health policymaking with equal opportunity for representation at policymaking levels as other health care professions.

*“We are pushing for pharmacists to get more into the public sector with policy elements, because if you don't have pharmacists involved in policy developments and policy discussions. This will ensure that the role of pharmacists is appreciated across the board...”* East-Central Africa—C6

Councillors felt that the laws and policies to support and empower the role of pharmacists are currently unclear in some Commonwealth nations. Even if there is a law supporting and progressing pharmacy profession, the enforcement and implementation of such laws remains weak. The councillors indicated that the absence of pharmacist representation in laws and policy making at Government or Ministry level, have caused the national laws to be insufficient, undefined, and unrestrictive towards non-registered and fake activities related to pharmacist, medicines and medicines services.

*“We need advocacy (pharmacist) on comprehensive laws that will help guide the pharmacy space. As we are in our country today, the laws are not sufficiently registered to prevent interlopers for pharmacists or fake (and) non-registered activities (medicine services). We have issues with policies and policy implementation”* Western Africa—C4

#### (c) Development of collaborations to support capacity building and health systems strengthening

Councillors indicated that the pharmacy profession is not yet recognised for its professional capabilities due to lack of expertise regarding skills of pharmacists and a lack of pharmacist advocacy. Councillors strongly emphasised that there is a need to increase the capacity building of pharmacists and to prioritise the strengthening of the profession by developing interdisciplinary collaborations, networking, and supportive initiatives at national and international levels. Many councillors from African nations felt a strong need to establish external (foreign) collaborations to help develop and promote the pharmacists’ professional capacity, scope, and skills in respective domains.

*“In most countries the pharmacy profession is not recognised. We still need some expertise from collaborative networks in a lot of areas of practise, and responsible drug use…”* Southern Africa—C5

*“Personally, clinical practice of the pharmacists will benefit from partnerships…if a particular pharmacist from a foreign country can come and work with the hospitals and show them (the) ideal work by pharmacists, so they (then) learn how to set up systems. Despite all the work, they have to do. Sharing this knowledge will be good...”* East-Central Africa—C6

Another common barrier perceived by councillors was that pharmacist in LMICs are still used for medicine dispensing, stocking, and inventory control only. Councillors felt that pharmacists in LMICs are being under-utilised for providing patient-centred services including medication management and rationalisation services. The councillors perceived that international organisations such as FIP and CPA could help support NPAs in providing strong advocacy to develop advanced clinical pharmacy practitioner roles and provide evidenced-based practices to evaluate the impact and outcomes of these services.

*“Many (policy makers) are still pigeon-holing us into dispensing medicines, attempts to get into the clinical pharmacy areas is simply resisted in some areas and we need bilateral support from CPA and FIP in these areas to show that clinical pharmacy is not a local idea. It is a global idea that serves citizens and health care demanders (public) well. Those are greater areas we need advocacy in...”* Western Africa—C4

Councillors also shared that international medicine formularies can sometimes be lacking in context for individual nations. They felt that international organisations, such as CPA, could help them develop national medicine formularies that should be formulated based on respective national needs.

*“Raising what pharmacists can do and educating the public on that... including a national formulary that reflects local medication and guidelines. We need (CPA) support for the increased role in pharmacy...”* East-central Africa—C7

Developing national pharmacovigilance centres and connecting them to Uppsala Monitoring Centre (UMC) and WHO to help support allied health professions in promoting patient safe use of medicines were also found to be a common priority across Commonwealth nations. The councillors indicated a need to develop technical infrastructure and databases to help facilitate these centres and monitor a sustainable outcome of safe medicine use.

*“The Ministry of Health (MOH) is working with WHO pharmacovigilance centre in the Uppsala Monitoring Centre (UMC) for the drug safety and international drug monitoring through VigiBase®, for adverse drug reaction (ADR) reporting. This needs more technical infrastructures and improvement to be able to keep on”* North America—C8

Support from external organisations such as CPA was also considered essential for mentorship to develop young pharmacists’ career pathway guidance in Commonwealth countries.

*“It is important to develop shadowing and mentorship from CPA international pharmacists in the UK to pharmacist practitioners and clinical pharmacists in Commonwealth nations, to give them more opportunities to connect with other role model pharmacists, meet periodically, share experiences and discussion to improve their practice”* Western Africa—C9

Councillors perceived that an integrated interdisciplinary heath care approach could have positive effects in knowledge and skills exchange across health care teams in various public health issues.

*“Collaboration is key, we can't do this alone, there has to be lots of planning, benchmarking from others. Once we have the collaboration aspects of education, regulation, where we pharmacy (NPAs) could be involved, (this) would tie into that, to ensure that we have a well set up machine (workforce) that provides quality pharmaceutical delivery...”* East-Central Africa—C6

#### (d) Creating opportunities for extended roles and revising remuneration

Most of the councillors from Asian and American nations perceived that the current pharmacy education and job markets across some Commonwealth nations are not coherent. There is a lack of practice-based job opportunities for pharmacists that leads to insufficient usage of their unique skills and position in society.

*“Although they are being trained, there are not enough jobs for pharmacists in the community and hospital to actually practise in the way they’ve been trained”* South East Asia—C9

*“The pharmacists don’t mix the IVs in the local hospital, the nurses do…So we definitely have a shortage of pharmacists in these roles”* The Americas—C10

Councillors from both Asian and African countries presented their views that they want to prioritise on having proper remuneration models and payments for pharmacist services. Not getting enough payment could become a potentially negative determinant for non-motivation of pharmacists to provide patient centred services.

*“There is no dispensing fee or professional fee. We (NPAs) have asked for it, and we keep asking. We want to have more remunerations (for pharmacists) so that at least when they finish for the whole day, they know they are getting a decent salary”* East Africa—C11

*“There is a problem of remuneration. The pharmacists are not motivated to start working on patient side of things instead of regulatory (dispensing) work because pharmacists are not part of a system that is designed appropriately.”* South East Asia—C9

#### (e) Achieving UHC goals

NPAs from both Asian and African countries shared their priorities for the development of pharmacy services in the health care systems and wanted to provide evidence about the contribution of pharmacists to achieving sustainable UHC goals. Councillors mentioned that realising the impact of pharmacy services and the added value of the pharmacist would pave the way of developing pharmacy workforce and advanced services.

*“If we can start showing that we have enough information and understanding, we can save enough money and aid the health of the patients for universal health coverage”* Southern Africa—C16

*“We can provide universal health coverage, many centres where the public can achieve health care and awareness”* South Asia—C15

One of the main goals of UHC is to provide access to medicines; however, the pricing of new medicines from authentic suppliers remained a huge barrier for NPAs in Commonwealth nations.

*“One of the concerns is to be able to access quality pharmaceuticals at affordable prices to get it when they need it. “*The Americas—C12

Councillors from African and the American Commonwealth nations mentioned that they encounter substandard and falsified medicines in the market, which comes a huge barrier in fulling UHC goals of genuine and quality medicines.

“*Substandard falsified medicines are also still an issue, there are still counterfeits and false unlicensed sellers”* South East Africa—C13

*“The other area is about drug training and quality assurance. As (well as) to deal with counterfeits.”* The Americas—C14

## Discussion

This is the first study to identify gaps and priority areas for NPAs across Commonwealth LMIC nations and to investigate the priorities around developing capacity in the pharmacy profession to help provide strengthening to their unique health care systems for achieving UHC and SDGs goals. This is also the first study to report on pharmacy profession needs and priorities in LMICs, where data are often lacking Three main overarching priority areas; extending pharmacy services, improving pharmacy education and redefining the role of NPAs have been identified by the councillors as their priorities to develop or to improvise pharmacy profession to achieve UHC and SDG goals in respective countries.

Extending pharmacy services was one of the major areas of focus identified by the Commonwealth nations in this study. Nearly all of the Commonwealth nations expressed a need to enhance community pharmacists’ roles to provide advanced patient oriented medicine services as they have the potential to provide health care advice to all categories of people [30]. Evidence shows that the services by pharmacists in community pharmacies help to reduce overall health care costs by lowering the occurrence of expensive forms of treatment such as hospital admissions and emergency room visits which may occur due to inadequate medicine use and/or adverse reactions [31]. Although the potential for pharmacy profession to enhance its contribution to health care is widely recognised in high income countries [32, 33] the evidence across LMICs is scant [27, 30, 34]. Similar to our results, other individual studies have also identified the need to develop community pharmacy services in LMICs [35, 36]; however, countries across Commonwealth nations have their own priorities in health policy reforms, health care systems, pharmacist based services’, and socio-cultural contexts affecting the development and implementation of services in community settings [37]. Although these varying features seem to cause barriers to NPAs to achieve UHC goals, they can be also considered as opportunities for future service development in different countries and settings. An overarching body such as CPA could take on the responsibility and take certain initiatives to guide and support NPAs through their journey to developing and implementing advance community pharmacy services to achieve UHC goals uniformly across Commonwealth nations.

In our study, most of the Commonwealth nations mentioned that clinical hospital-based pharmacy services should also be developed to improve patient outcomes which is consistent with previous studies [35, 38]; however, many councillors also felt that there is currently an excessive deficiency in clinical pharmacy services in Commonwealth nations. This insufficiency might be due to the challenges with regards to pharmacists’ education and training across LMICs resulting in insufficient clinical skills along with a lack of opportunity to provide patient-oriented pharmacy services [34, 39]. Due to insufficient involvement and opportunities for clinical pharmacists, the councillors said that the burden of provision of optimum patient services falls on other healthcare professionals such as nurses and paramedics burdening overall health care systems. Interdisciplinary team based care has the potential to improve medication use and reduce adverse drug events and cost [40]. However, our interviewees identified a lack of interdisciplinary collaborations. As part of CPA’s mission strategies in aiding NPA’s overcome this barrier going forward would require deep understanding and emphasizing the processes and advantages of well-functioning multidisciplinary teams across LMICs and the added value that pharmacists can provide in complex health care systems by elevating pharmacists’ professional skills and capacity.

This is in line with the second theme, where most interviewees expressed a need to improve initial and postgraduate pharmacy education, building to support extended pharmacy services. There is a need to launch CPD programs with throughout the Commonwealth nations to improve the knowledge, capacities and skills of pharmacists. According to FIP’s report on pharmacy education in sub-Saharan Africa [41] a lack of a competent pharmaceutical workforce hinders achieving the concept of pharmaceutical care in Africa. This could be due to lack of advanced clinical knowledge and skills due to a deficiency in the pharmacy curricula. The curricula in most Commonwealth nations are focused on non-clinical training and presents a major challenge to upskill pharmacists to perform advanced roles [41–44]. The deficiency, however, cannot be fulfilled just by upgrading the curricula with clinical components, because academics teaching pharmacy curricula are deficient and not adequately trained to teach advanced clinical skills. Improving the skills and knowledge of academics teaching the pharmacy curricula could facilitate the delivery of a clinically focussed curricula in Asian as well African Commonwealth nations [42]. Pharmacy education and specialised training would be an essential building block to build a competent workforce which can hopefully improve and empower pharmacy professionals to achieve UHC and SDG goals. The CPA has an increasing number of works streams and is probably best known for its highly valued PharmAid scheme that has (since 1970) redistributed recent versions of medicines information resources such as the British National Formulary from the NHS to LMICs in the Commonwealth in response to requests from members. Alongside PharmAid, joint biennial conferences have been the other longstanding activity that the CPA has been associated with. The CPA pioneered and is the technical delivery partner for the Commonwealth Partnerships in Antimicrobial Stewardship (CwPAMS) programme across 4 African countries. Through current work in Ghana, Tanzania, Uganda and Zambia to strengthen AMS, the CPA has conducted an ongoing needs assessment and has identified the need to build capacity in pharmacists already engaged in the scheme, to disseminate antimicrobial skills and knowledge for more widespread impact. The charity is currently developing its online and continuing education offering, and recently launched its online education platform to support CPD through the NPAs. The CPA has also led a range of international workshops and events that are aimed at upskilling the pharmacy workforce and providing resources for pharmacists to help them improve their practice. Despite all these efforts by CPA, this study identified that currently the LMICs in Commonwealth nations are struggling to improve and raise their professional deficiencies due to a lack of a professional body as well as an independent pharmacy regulator and a medicine regulator.

Most of the Commonwealth nations do not separate and segregate professional regulation and medicines regulation. This study highlights the vital need for segregation or redefining the core responsibilities and/or reassigning tasks of a pharmacy independent regulator, professional body and medicines regulator [45]. Improving pharmacists’ core skills, developing new practice based roles, sustainability and the impact evaluations of these roles falls under the domain of NPAs, whereas issue about medicines such as pricing, distribution, access to medicines, import and export of medicines are the specific roles of medicine regulator [46, 47]. It is also imperative to empower these roles with the support of local laws and regulations. Having this segregation in LMICS might help improve the individual capacities of these organisations to effectively plan, design focused strategies and to execute implementation of future services to achieve these two different roles [48, 49]. This would help strengthen the health care systems to achieve UHC and SDG goals in Commonwealth nations [50].

It was also identified that there is an urgent need to develop, restructure, and redefine the role of professional bodies, to provide support to pharmacists, improve the pharmacy profession and represent pharmacists in national policy making. There is also a need to develop and improve professional practice, design competency frameworks, develop guidelines to improve practice and support for pharmacists looking to develop/improve pharmacy services [51]. This might empower the pharmacy profession to help achieve a pathway to support UHC and SDG goals. The councillors also identified the need to have an independent pharmacy regulator to allow a focus on the development and enforcement of standards for the profession, distinct from other health professionals. In addition, a need was identified to establish a separate medicines regulator that could focus on medicine-related issues and ensure adequate supply of essential medicines and enable access to quality medicines [52, 53].

Despite the immense advocacy opportunities that NPAs can play in terms of achieving and improving public health benefits as well as progressing the pharmacy profession, in most Commonwealth nations are not usually involved in national strategies [54, 55]. Therefore, NPAs gaining a statutory recognition and gaining representation at the national level has become a recent priority for LMICS amongst Commonwealth nations. However, NPAs strongly perceived that they need international organisations like FIP and CPA to help them advocate for their interests, help evaluate the impact and present their policy recommendations to governments. The interviewees also felt that supporting and networking between NPAs across Commonwealth nations was extremely important to align national interests across LMICs and play their part in the achievement of UHC and SDG goals [56].

The response of the global pharmaceutical workforce to the COVID-19 global pandemic has been remarkable [57]. The Commonwealth NPAs have been currently providing support to minimise the impact of COVID-19 to the public, especially by managing chronic conditions and ensuring medicines supply, when many other health care facilities were overwhelmed, emphasising the vital role of community pharmacists as accessible healthcare providers.

The response of the pharmacy profession to the pandemic has demonstrated important and diverse opportunities to expand the role of pharmacists beyond conventional roles to facilitate and provide better access to medicines and medicine related health services.

New policies and laws may be needed to support the evolution of pharmacy practice and to support this governance, for example the establishment of senior pharmacist in advocacy roles at government level, and involvement of NPAs in the creation and implementation of health policies [19]. Pharmacists should be key stakeholders in all discussions involving medicines if systems and policies are to be well thought out and effective [58]. Governments need to develop pharmacists’ skillsets and take appropriate steps to strengthen the profession and ensure pharmacists are appropriately regulated and recognised [48, 49]. This good governance facilitates better regulation of medicines, pharmacists, and pharmacies. Without legislation or governance that specifically defines pharmacist roles and pharmacy practice, it is difficult to advocate for the extending role of pharmacists. In parallel, more evidence for the impact and value add that pharmacists bring to multidisciplinary teams and services, particularly in LMIC settings is needed to facilitate this change. There is potential for the CPA to support this by creating online knowledge sharing symposia, webinars, CPD, and online workshops [48, 58].

This study presents a summary of the NPAs councillors views and their focus to prioritise the upscaling of the capacity of pharmacy workforce in Commonwealth nations as well as a need to develop and involve NPAs in national strategies to utilise pharmacy workforce to achieve UHC and SDG goals [59]. The magnitude of scope of this study and the outreach activities of this study might not just be important for CPA strategic planning of supporting member organisations but also for NPAs aligning and revisiting their national strategies to help achieve UHC and SDG goals. It was interesting to find that there may be different pharmacy regulatory models and regulators across commonwealth countries and their role and capability may be contextually dependant on many intrinsic and extrinsic factors of respective governments as well as pharmacy representative bodies. These contextually varying determinants could have given rise to varying states of advancement of the pharmacy profession across commonwealth countries as well as the capability of NPA’s to advocate for the upgradation of pharmacy profession. Future studies should look at the different regulatory models currently in practice across commonwealth countries and their role and impact on pharmacy profession and pharmacists.

## Strengths and limitations

The major strength of this study is that it used an exploratory qualitative methodology which given the range of participants throughout Commonwealth nations allowed a flexible format and helped develop understanding and gain participants’ trust and allowed the authors to explore participants’ responses in-depth [25]. This approach provided the interviewers with enough flexibility to explore emergent lines of inquiry while also keeping the interview focused on the aim of this study. Another major strength of this study is interviews with participants who work directly ‘on the ground’ and know what the current landscape is and can be improved and provides a realistic need base assessment for health needs.

One of the limitations in this study was that all three authors involved in the analysis were pharmacists by background, so it was impossible not to have any preconceptions. However, the authors attempted to remain reflexive and acknowledge their own perceptions and approach the research without having any apriori assumptions. To further minimise the researcher own assumptions and bias, the reflexive, thematic analysis method helped in seeing the data neutrally and the themes were inductively identified after a rigorous discussion. In addition, using an LMIC expert (RE) who was oblivious to the data set and the project, to check the coding and themes, provides rigour in analysing the data without having any strong expectations about steering the development of pharmacy profession across commonwealth nations, respectively, in any direction. Another limitation of this study is that participants from LMICs which did not respond to the invitation could have added some new directions or priorities for NPAs for achieving UHC goals; however, using a constant grounded theory comparison analysis method to reach “saturation” overcomes that limitation to a certain extent [60].

## Conclusion

Pharmacists are still an underutilised healthcare resource and there is a critical need to focus at the national as well as global level for pharmacy professional services to be developed, implemented and utilised in their full potential in all LMICs including Commonwealth nations. This study summarizes how the NPAs across the Commonwealth have identified their priorities and are planning to improve or develop the pharmacy profession to strengthen health care systems in their respective nations. Understanding the priorities; extended pharmacy services, improved pharmacy education and standards and developing and redefining the roles of professional leadership bodies, will help CPA develop tactical planning strategies to support member organisations in achieving UHC and SDG goals in the future.

## Data Availability

The data that support the findings of this study are available from Commonwealth Pharmacist Association, but restrictions apply to the availability of these data, which were used under license for the current study, and so are not publicly available. Data are, however, available from the authors upon reasonable request and with permission of councillors.

## Abbreviations

ADR: Adverse drug reaction
AMR: Antimicrobial resistance
C: Councillors
CHPA: Commonwealth Health Profession’s Alliance
CPA: Commonwealth Pharmacists Association
CPD: Continuous professional development
CwPAMS: Commonwealth Partnerships in Antimicrobial Stewardship
FIP: International Pharmaceutical Federation
GDPR: General data protection regulation
LMICs: Low and medium-income countries
MOH: Ministry of Health
NPA: National Pharmacy Associations
NCDs: Non-communicable disease management
SDG3: Sustainable Development Goal-3
SDGs: Sustainable development goals
UHC: Universal health coverage
UMC: Uppsala Monitoring Centre
WHO: World Health Organization
